# Improvement of bioprocess monitoring: development of novel concepts

**DOI:** 10.1186/1475-2859-5-19

**Published:** 2006-05-22

**Authors:** Franz Clementschitsch, Karl Bayer

**Affiliations:** 1 Baxter Bioscience Research Center Orth an der Donau, Austria; 2 Department of Biotechnology, University of Natural Resources and Applied Life Sciences, Vienna, Austria

## Abstract

The advancement of bioprocess monitoring will play a crucial role to meet the future requirements of bioprocess technology. Major issues are the acceleration of process development to reduce the time to the market and to ensure optimal exploitation of the cell factory and further to cope with the requirements of the Process Analytical Technology initiative. Due to the enormous complexity of cellular systems and lack of appropriate sensor systems microbial production processes are still poorly understood. This holds generally true for the most microbial production processes, in particular for the recombinant protein production due to strong interaction between recombinant gene expression and host cell metabolism. Therefore, it is necessary to scrutinise the role of the different cellular compartments in the biosynthesis process in order to develop comprehensive process monitoring concepts by involving the most significant process variables and their interconnections. Although research for the development of novel sensor systems is progressing their applicability in bioprocessing is very limited with respect to on-line and in-situ measurement due to specific requirements of aseptic conditions, high number of analytes, drift, and often rather low physiological relevance. A comprehensive survey of the state of the art of bioprocess monitoring reveals that only a limited number of metabolic variables show a close correlation to the currently explored chemical/physical principles. In order to circumvent this unsatisfying situation mathematical methods are applied to uncover "hidden" information contained in the on-line data and thereby creating correlations to the multitude of highly specific biochemical off-line data. Modelling enables the continuous prediction of otherwise discrete off-line data whereby critical process states can be more easily detected. The challenging issue of this concept is to establish significant on-line and off-line data sets. In this context, online sensor systems are reviewed with respect to commercial availability in combination with the suitability of offline analytical measurement methods. In a case study, the aptitude of the concept to exploit easily available online data for prediction of complex process variables in a recombinant *E. coli *fed-batch cultivation aiming at the improvement of monitoring capabilities is demonstrated. In addition, the perspectives for model-based process supervision and process control are outlined.

## Introduction

Bioprocess technology is currently employed for the production of several economically important commodity and fine chemicals [1], enzymes and therapeutically active recombinant proteins. To give an indication of the market volume of the production of recombinant proteins, the top selling biopharmaceutical product of the year 2000, reached a world sales volume of € 13 billion in [2]. Due to economic needs and because of the complex nature of microbial growth and product formation in batch and fed-batch cultivations, the monitoring and control of bioprocesses represents an ever-increasing engineering challenge. In order to achieve optimal exploitation of the particular production organism, the advancement of bioprocesses to improve monitoring and control capabilities is pivotal to achieve reduction of production costs and increase of yield while at the same time maintaining the quality of the individual metabolic product. However, a great number of the current processes are still far from being optimal mainly due to limited process monitoring capabilities.

For further optimisation of bioprocesses as well as for ensuring high, consistent product quality, the lack of accurate real-time monitoring of different physical, chemical, and biological parameters will be the bottleneck. Therefore, in the years to come, increasing focus has to be given to online/inline techniques for process monitoring, driven by the industry's never-ending need for process optimisation. Further requirements will arise by regulatory affairs like the process analytical technology (PAT) initiative issued by the FDA. The major goal of PAT is to improve the understanding and control of the manufacturing process: quality cannot be tested in products, it should be built-in or should be achieved by design. Process Analytical Technology is seen as a system for designing, analyzing, and controlling manufacturing through timely measurements (i.e. during processing) of critical quality and performance attributes of raw and in-process materials and processes with the goal of ensuring final product quality. The observation of variables related to the biological system, representing the production entity, is one of the key requirements to enable controlled gene expression and optimal operation of the host cell. On the contrary, key variables of cultivation processes are still beyond direct measurement despite the progress in monitoring and control of bioprocesses in the last years [3]. A direct reading of biological key process variables, such as biomass, has not yet been achieved, although online sensor systems that provide different types of signals are available. However, the inability to directly measure these key process variables does not imply one cannot extract valuable information from the bioprocess. In order to develop novel monitoring concepts, it is necessary to scrutinise the role and properties of the different cellular compartments to the synthesis process of economically important biotechnological products at first.

## Cellular compartmentation in the context of the synthesis process of economically important products

**Figure 1:**
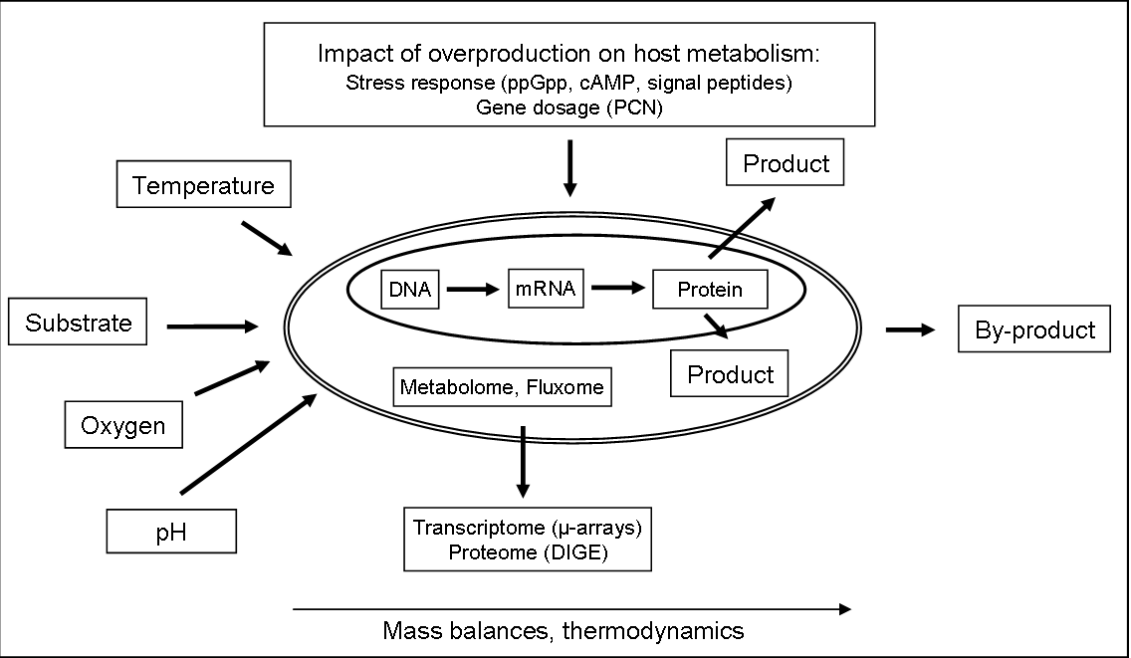
The production of economically important biotechnological products is governed by a multitude of influencing factors hat require a multidisciplinary approach (biochemistry, molecular biology, analytical methods as well as mass balances and thermodynamics).

The principal goal of biotechnological production of economically important substances is in most cases a maximisation of space/time yield. However, the desired overproduction of a given substance of interest most likely affects the cellular metabolism in many ways as the cellular metabolism is a tightly controlled and highly interconnected network. Therefore, aiming at optimisation, the role of the different cellular compartments in the biosynthesis process must be taken into account. Consequently, analytical methods must be available to identify and, in a second step, allow the online monitoring of these variables during a cultivation process. A schematic illustration shows the variety of variables that must be acquired and monitored during the cultivation process (Figure 1).

Consequently, a variety of complex key process variables of the host and, in the case of recombinant protein production the expression vector system needs to be monitored, as well. In the case of recombinant cultivation processes, relevant variables can be divided into two groups:

• Variables related to properties and functionality of the host cell system, comprising biomass concentration, cell number, cell viability, metabolic stress response, specific quantities of metabolites and activity of particular entities.

• Variables related to the applied expression vector system, comprising the recombinant gene dosage (PCN), content of recombinant product and the product formation rate.

In order to adjust product formation rates, in particular recombinant gene expression, in relation to the metabolic capabilities of the host cell's synthesis machinery, real-time knowledge of a cluster of key process variables over the whole process is of paramount importance. Hence, online monitoring methods are required providing specific knowledge for optimal process operation, e.g. maximal exploitation of the host cells synthesis capacity or termination of the cultivation process.

## Monitoring of biotechnological cultivation processes

The current state of bioprocess monitoring has evolved from chemical engineering. In general only a low number of variables can be acquired followed by thorough characterisation of the product. By maintaining environmental conditions within validated limits it is "assumed" that the product is formed according to defined regulatory issues. The current monitoring concepts are developed due to the lack of sensors not considering the strong dynamics of the biochemical synthesis process in combination with its complexity. With regard to process monitoring it is important to have in mind that process development and optimisation of a novel product requires the observation of many more variables than in a validated production process.

Therefore in the first instance the development of novel bioprocess monitoring concepts has to identify significant monitoring and/or control variables strongly involved in pathway regulation and regulatory networks. In the next step the information contained within individual data sub-sets, i.e. the potential contribution to improve process knowledge must be assessed, whereby as many as possible on-line and in situ signals should be acquired. However, the availability of on line sensors useful for in situ bioprocess monitoring is comparatively low due to specific requirements of aseptic conditions, high number of analytes, drift, and often rather low physiological relevance. Sensors used in situ must not contaminate the bioprocess and it is obvious that their components must not leak into the culture (biocompatibility). Additionally, sensor systems must be able to perform without recalibration as bioprocesses may potentially run for weeks. A comprehensive overview over today's instrumentation of bioprocesses is given in [4].

## Measurement principles and commercial availability of state-of-the-art online sensor systems

In the following paragraph, state-of-the-art online sensor systems are described that have the potential to yield high-quality signals from a bioprocess.

The measurement principle, delivered information and examples of state-of-the-art sensor systems commercially available for bioprocess monitoring, advantages and disadvantages are presented (Table 1). A direct assignment of a signal to a biological variable is not possible, except the Biomass Monitor, were capacitance can be allotted to biomass.

**Table 1:** 

**Measurement principle**	**Target/Information**	**Vendor (commercial system e.g.)**	**Advantages**	**Disadvantages**
Paramagnetism	Oxygen/mass balancing	ABB Ltd CH-8050 Zurich Switzerland	Selectivity, stability	Desiccation of sample, delayed delivery of representative gas sample due to varying head space
Dielectric spectroscopy	Membrane-enclosed biovolume	Aber Instruments (Biomass Monitor)	Good correlation to biomass	Signal influenced by variation of conductivity of fermentation broth
2D-Fluorescence spectrometry	Typical intracellular substances involved in metabolic pathways	DELTA (BioView)	Capture of minute changes in chemical composition of the cell	Direct reading of process variables not possible multivariate data analysis required
Infrared spectrometry	Typical intracellular substances	FossNIR-Systems (Model 6500) ABB (BOMEM MB160)	Spectral fingerprint of principle cellular constituents	Direct reading of process variables not possible multivariate data analysis required
Mass spectrometry	Volatile organic compounds	Ionimed (PTR-MS)	Identification of chemical components, mass balancing enabled	Critical issue: sampling of head space
Metal Oxide Field Effect Transistor	Volatile organic compounds	ALPHA M.O.S	Versatile sensor arrays	Direct reading of process variables not possible multivariate data analysis required

The price/performance ratio of the above listed devices varies from approx. 20 k€ for e.g. oxygen analysers to about 50 k€ for e.g. optical and dielectric spectroscopy and finally up to 100 k€ for highly sensitive mass spectrometry based systems.

Widely applied in bioprocess monitoring are spectroscopic methods, which are based on the interaction of a sample with electromagnetic radiation. These versatile techniques are well suited to capture changes in bioprocesses, as spectroscopy allows non-invasive, non-destructive and continuous monitoring of a process.

Dielectric spectroscopy makes use of the electrical properties of the cells, which are exposed to a radio-frequency electrical field and has a high potential to provide online monitoring of cellular properties [5]. Cells with intact plasma membranes act as capacitors, since the non-conducting nature of the cell plasma membrane allows the build up of charge. The resulting capacitance is measured and is dependent upon the cell type, the cell size (as the size determines the cell volume) and the concentration of viable cells [6]. Although the above mentioned factors influence the capacitance reading, this method is less susceptible to particles or fouling effects than optical turbidity measurements. However, due to the underlying measurement principle, it cannot deliver signals regarding intracellular components and therefore insights into metabolic state and cellular metabolic activity are possible only to a very limited extent. Optical sensor systems for process monitoring allow non-invasive in vivo monitoring of bioprocesses, do not interfere with the metabolism of the cells and consequently offer versatile intracellular information that is nearly impossible to obtain with other methods. In addition, sampling (always a potential source of contamination) and sample pre-treatment are generally not necessary for optical sensor systems and in most cases no analyte is consumed [7]. Today, the available optical spectroscopy sensor devices for bioprocess monitoring use 2 different regions within the electromagnetic spectra. Within the visible part of the electromagnetic spectrum, fluorescence spectroscopy is a useful tool particularly since the development of multi-wavelength devices in the early 1990's [8] due to the fact that many intracellular components show fluorescence properties. Former online fluorescence sensors used only one excitation and one emission channel, limiting the sensor to monitor a single fluorophore. Recently developed fluorometers use several excitation and emission wavelengths, increasing the number of constituents in the biosuspension that can be monitored simultaneously [9]. Multi-wavelength fluorescence spectroscopy provides direct monitoring of changes in biologically relevant fluorophores, e.g. NAD(P)H, which can provide information about the energetic state of the cell and the state of oxygen supply, in particular close to anaerobic conditions. As many of these fluorescent compounds play crucial roles in metabolic pathways, this technique has proven to be a valuable tool for bioprocess monitoring [10,11].

Outside the visible range of the electromagnetic spectrum, near infrared light (NIR), ranging from 780 nm to 2526 nm (12820 to 3959 cm-1, as defined by the American Society for Testing and Materials), can be used to measure the concentration of certain organic species, even in complex media. Biologically important bonds (aliphatic C-H, aromatic or alkene C-H, amine N-H and O-H) absorb in the NIR range. Each chemical structure is related to a specific position, shape, and size of the analyte's absorption bands. Process related changes could be captured in the NIR spectra of the culture fluid [12] and infrared and Raman spectroscopy were applied to online bioprocess monitoring [13-16]. For further reading please refer to [17].

**Figure 2:**
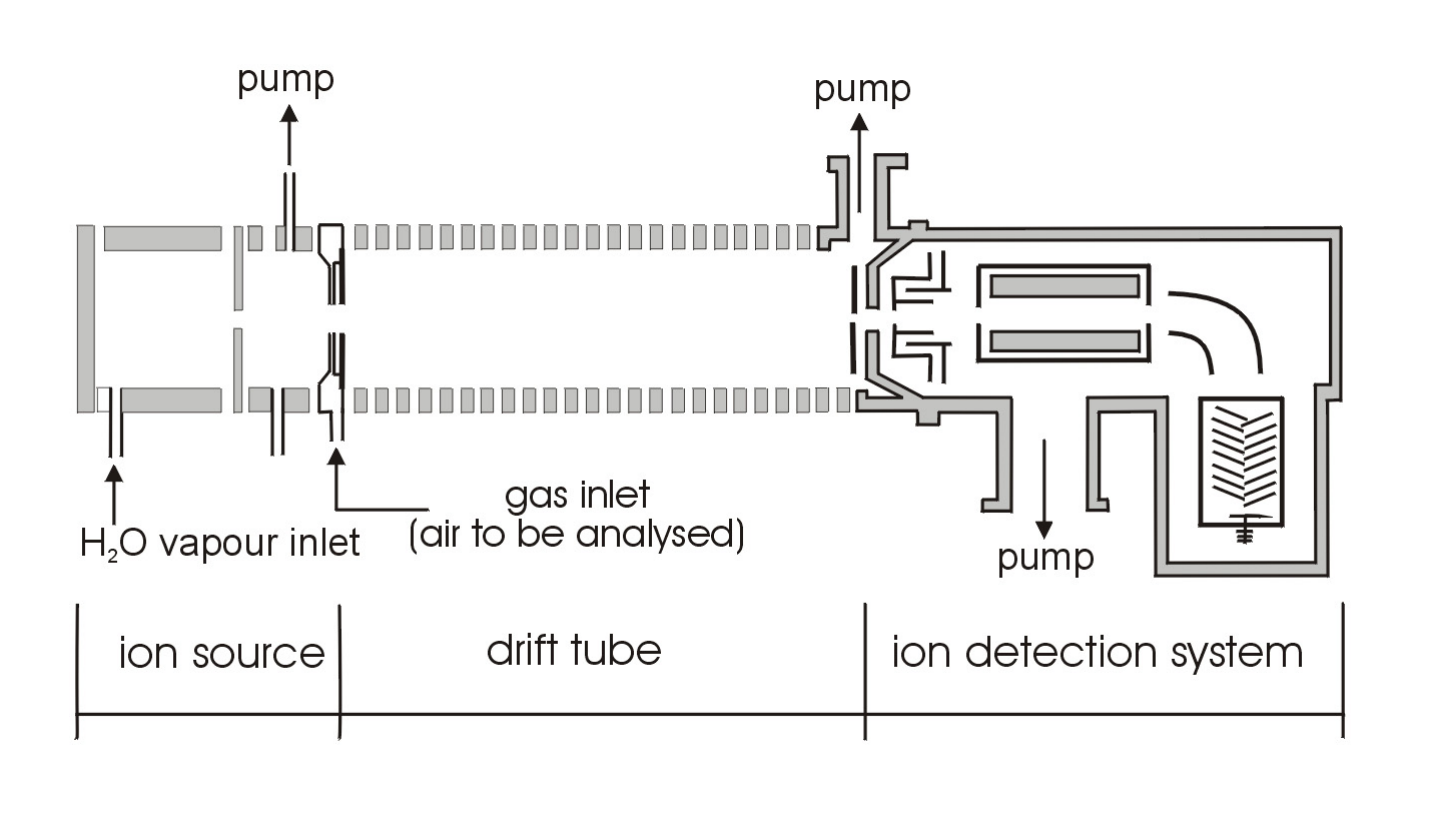
Schematic representation of the PTR-MS apparatus [22].

Beside techniques that directly interact with the cells itself, methods that use process exhaust gas have similar advantages (non-invasive and non-destructive) and yield valuable information about the process. Chemical multi-sensor arrays (electronic nose) and mass spectrometry were applied to analyse off-gas of cultivation processes and have shown their aptitude for process monitoring [18-21], although the chemical multi-sensor array was never commercialised for the application as a sensor device for bioprocess monitoring purposes. In this context, it must be mentioned that, an electronic nose is available from the company ALPHA M.O.S., however, to the best of our knowledge, this system was not applied for bioprocess monitoring so far. Another commercially available system that allows the detection of volatile organic compounds (VOC) is Proton-Transfer Reaction mass spectrometry (PTR-MS). As a comprehensive description of the development of the PTR reaction principle can be found in [21], only a basic description of the reaction principle is given here: In a PTR-MS, primary ions H_3_O^+ ^react with uncharged molecules under well defined conditions. On the way through a reaction region (flow-drift tube prior to the MS-inlet) the ions perform many non-reactive collisions with buffer gas atoms or molecules (Figure 2). However, once they collide with a reactant gas particle they may undergo the following reaction: A^+ ^+ R → products. In the case of H_3_O^+^, these perform proton transfer reaction (if energetically allowed): H_3_O^+ ^+ R → RH^+ ^+ H_2_O.

PTR mass spectrometry allows the detection and quantification of compounds in the range of ppb to ppt. It has been applied to a variety of analytical tasks, such as medical applications via breath analysis, food research or environmental monitoring [23-25]. For example, the volatile organic compounds acetone, ethanol, methanol, propanol and isoprene were analysed in human breath, while in food analysis, methanethiol and dimethyl sulphide emanating from meat were monitored.

The aptitude for bioprocess monitoring was proven in our lab by chemostatic cultivations of *E. coli *where typical metabolic VOCs were identified (data not shown). Remarkable was the detection of sulphur containing compounds at the end of batch cultivation simultaneously to cell lysis (confirmed by flow cytometric analysis). Altogether the results so far make PTR-MS a promising technique for bioprocess monitoring.

## Mathematical methods for deconvolution and extraction of relevant information from large multivariate datasets

**Figure 3:**
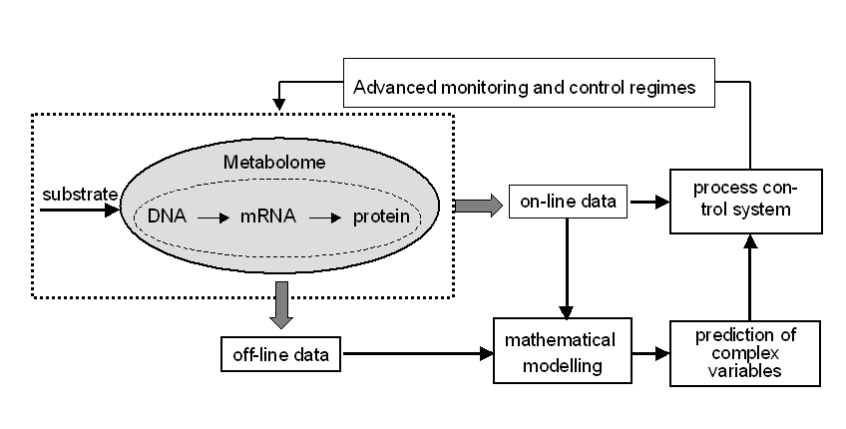
Integration of mathematical methods for advanced process control (adapted from [26]).

A common problem of the mentioned sensor systems is that the data do reflect changes in the process, but cannot directly be assigned to a biological process variable. However, the inability to directly measure key process variables does not mean that no relevant information can be extracted from process data. In this context, mathematical methods provide tools for structural understanding, exploratory simulation, interpretation, and evaluation of measured data or prediction and design. Thus, they assist to bridge the gap between online signals and the broad spectrum of complex offline data. Mathematical methods show a wide applicability and relevance for data analyses in bioprocess monitoring (Figure 3).

Mathematical models are indispensable tools to analyse, understand and control bioprocesses [26]. For process monitoring purposes, modelling allows the transformation of discontinuous signals into continuous variables. Several different approaches in model design, ranging from structured models (e.g. mass balances) to complex, unstructured nonlinear models like artificial neural networks, are described in [28]. This insight into a bioprocess is improved further if combinations of different online sensor systems are used simultaneously. Combining the data from sensors with different underlying measurement principles yields a complete picture, showing the process from different points of view and therefore holds great potential for process monitoring [29-31]. Today, chemometric methods are applied in two ways for bioprocess monitoring: i) to predict key process variables or ii) to visualise the course of cultivation by identifying the response patterns of the applied sensor systems. The term MSPC (Multivariate Statistical Process Control) was established to illustrate the application of multivariate methods for process supervision and control purposes. In an application of this approach, predictions of end-of-batch quality measurements were performed during the progress of a batch run [32]. Process monitoring, quality estimation and fault diagnosis activities are automated and supervised by embedding them into a real-time knowledge-based system (RTKBS). Albeit regulatory challenges, MSPC is not limited to academic or research purposes, the application of MSPC for an industrial penicillin process is described in [33].

## Case study: sensor combination and chemometric modelling for improved process monitoring in recombinant *E. coli *fed-batch cultivations

In order to demonstrate the aptitude of the aforementioned methods and concepts to improve process monitoring capabilities, a case study for recombinant *E. coli *cultivation processes is presented. For the production of a specific group of biopharmaceuticals, on lab and industrial scale as well, *E. coli *is an appropriate and widely used host due to its rapid growth and well-known physiological requirements [34-36]. Despite its importance, most current processes for production of recombinant proteins are characterised by a lack of process monitoring capabilities regarding biological key process variables. This limitation severely impairs the application of techniques for controlled expression of foreign genes in relation to the host cells metabolic synthesis capacity, such as transcription rate control [37]. For this approach, it is mandatory to obtain real-time information of these key process variables over the whole cultivation process. Aim of this case study was to improve process monitoring capabilities in recombinant *E. coli *fed-batch cultivations and to establish model-based process supervision and control in order to allow the full exploitation of the host cell synthesis capacity.

In order to extend the available signals for chemometric modelling, two specific sensor systems were chosen in addition to monitoring of standard variables (base consumption, off gas analysis). Dielectric spectroscopy was applied to obtain information regarding the host cell system, while two-dimensional, multi-wavelength fluorescence spectroscopy was applied as second sensor system to deliver signals regarding intracellular components.

**Figure 4:**
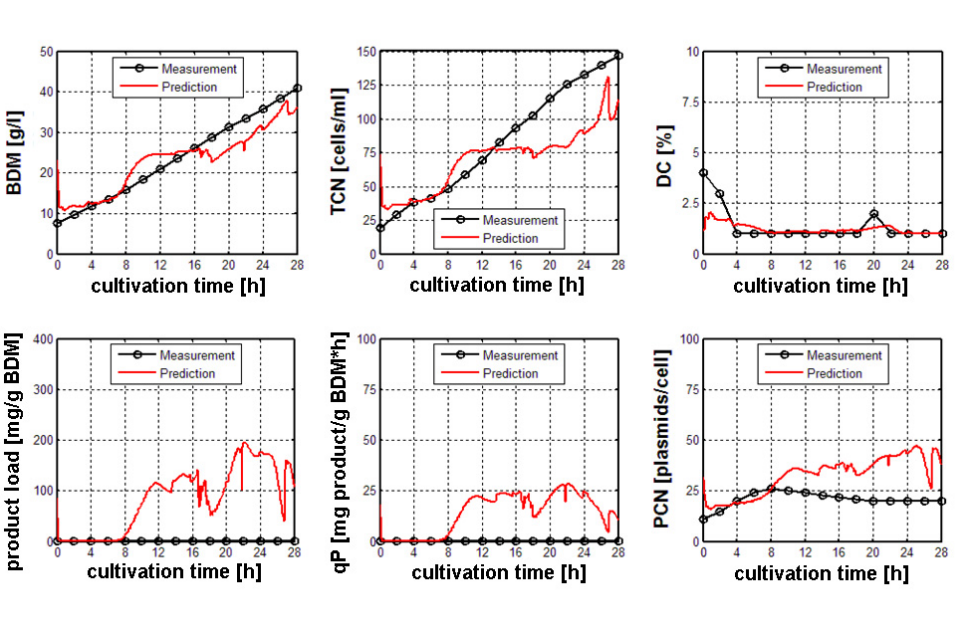
Prediction of target process variables BDM, TCN, DC, product load, qP and PCN of a non-induced cultivation experiment with a RBF model generated with classical input signals (CO_2_/O_2_, base consumption)

**Figure 5:**
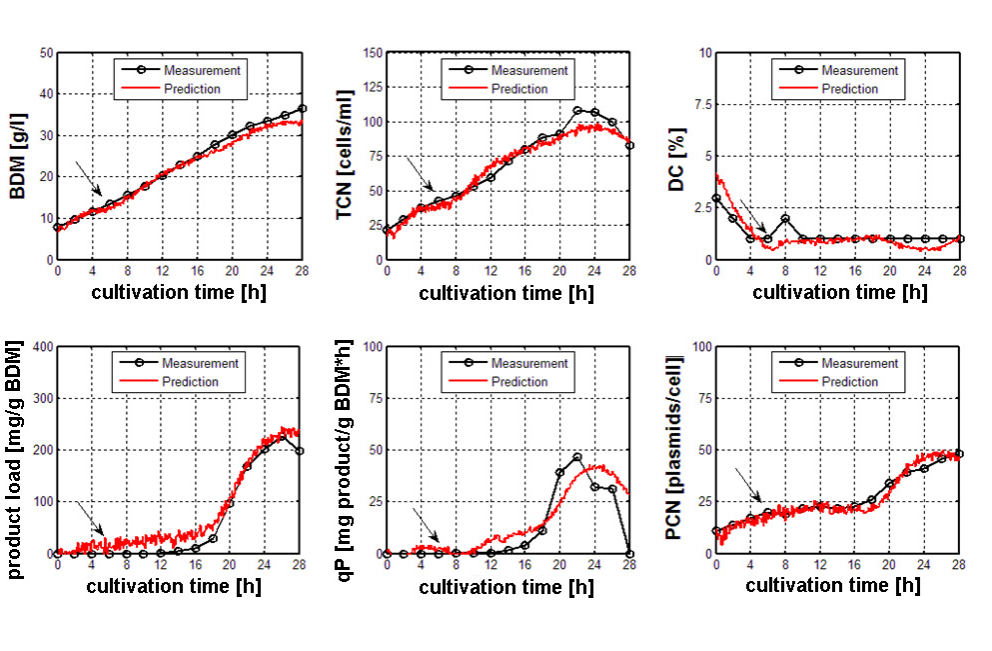
Improved prediction of target process variables BDM, TCN, DC, product load, qP and PCN with a RBF model generated with selected input signals (dielectric spectroscopy, fluorescence spectroscopy). Arrows indicate induction of recombinant protein expression.

Both sensor systems were applied in a series of identical fed-batch cultivations except different levels of recombinant protein expression in order to obtain appropriate data sets for training and evaluation. In total, 7 such cultivations were performed, whereby during each cultivation, the process variables off-gas composition, base consumption, capacity, conductivity and a complete set of fluorescence spectra were measured every 5 minutes. The cultivation was followed over 28 h, during the cultivation period the offline analytes biomass dry matter (BDM), total cell number (TCN), percentage of dead cells (DC), recombinant product and plasmid copy number (PCN) were determined. In order to induce recombinant protein expression IPTG was added to the bioreactor after 1 generation in the feeding phase in different ratios to biomass. Finally, for each cultivation a data set with 156 online signals and 6 offline target variables was obtained. The data sets of five cultivations were used to build multivariate process models in MatLab to estimate the offline target variables using two different chemometric algorithms, Partial Least Squares regression (PLS) and a Radial Basis Function neural network (RBF-NN). The predictive power of the model was evaluated against a data set previously unseen, i.e. not included in the training process. A comprehensive description of the experimental setup is given in [38]. It was shown that different sensor systems, different modelling algorithms and different input signals significantly influenced the prediction results. It was found that the best estimation results (i.e. the lowest error) for the estimation of the 6 target variables was obtained with a RBF network and selected input signals from both sensor systems. The improvement in estimation results is demonstrated in the figures 4 and 5, whereby bad performance of modelling due to unspecific on-line data is demonstrated by false positive prediction of product load and qP in a non-induced experiment (Figure 4). It was found that the lowest RMSEP's for all target offline variables were obtained when applying a non-linear RBF model that uses selected online signals of dielectric and optical spectroscopy as input (Figure 5). Regardless of the data set used for evaluation of the chemometric model, this approach yielded best results for all target offline variables, cell-related as well as product-related. In order to apply the previously created model in an online prediction during a cultivation process, a MatLab function was programmed to perform data input, chemometric modelling and display of the estimation results online during a cultivation process. Finally, a Graphical User Interface (GUI) was designed to facilitate the online estimation process and to prepare the application of this approach in further processes.

In this case study, it was demonstrated that online signals from different sensor systems in combination with chemometric modelling methods greatly extend the monitoring capabilities of complex biological process variables of recombinant host/vector systems.

However, the application of chemometric data analysis and model development for the forecast of important variables is not limited to recombinant protein expression or biotechnological processes. More examples for the successful application of MSPC can be found in [39], where 4 case studies are described using industrial cultivation data that utilise these models in the context of prediction and monitoring of bioprocess performance. In [40] online batch monitoring with dynamic PLS methods was performed in a chemical polymerisation process. Once established, model-based process supervision and control can greatly enhance and ease process supervision. In [41] real-time statistical process monitoring to an industrial *Bacillus *process was applied, where the operator is provided with a clear view of the process performance.

## Conclusion

In the years to come, increasing focus will be given to further exploitation of chemical/physical principles to enhance the spectrum of online/inline techniques and the application of miniaturised sensor systems, e.g. micro machines for process monitoring. This development is driven by the ever increasing needs to improve the efficiency of process development, to implement rational design and pursue the FDA's initiative regarding process analytical technology. Following the goal of PAT the manufacturing process must be better understood and easier to control. Quality cannot be solely derived from the products, it should be built-in or should be by design. Therefore Process Analytical Technology relies strongly on monitoring of physiological relevant variables, which have to be gained "on-line" by modelling. By application of the modelling approach the discrete offline samples are transformed into continuously available signals, whereby permanent supervision is provided and moreover deviations from predefined states can be identified at early stages. Within the PAT framework, multivariate data acquisition and analysis is an important tool, which highlights the upcoming importance of these methods regarding regulatory affairs. Gains in quality, safety and/or efficiency will vary depending on the product and are, among other points, likely to come from increasing automation to improve operator safety and reduce human errors. This implies that MSPC is going to be applied not only for monitoring, but more frequently for control purposes also. As an example, constituents predicted simultaneously in a *V. cholerae *cultivation have been used for automatic feeding control [42]. A key aspect of this work is the fact that the proposed control loop aided to keep the specific growth rate under the critical value, restricts further formation of acetate and thus consequently limits inhibitory effects of this by-product. Consequently, a possible future scenario regarding model based process supervision and control is given in [43]: More than 1800 different signals from gas sensors, electrodes, spectrometer detectors, balances, flowmeters, etc., were integrated in a data set and used for processing. By application of a number of computational tasks such as partial least-square regression, principal component analysis, artificial neural network modelling, heuristic decision-making and adaptive control the benefit of this concept was proven and demonstrated on different cultivation processes which illustrated sensor fusion control, multivariate statistical process monitoring, adaptive glucose control and adaptive multivariate control.

To achieve further advances in bioprocess optimisation, key process variables describing the potentials and limits of the biological system need to be available online. As it was shown, online signals from different sensor systems in combination with chemometric modelling methods allow the timely estimation of complex biological variables. This approach has the potential to enhance the process monitoring capabilities and to fulfil the upcoming requirements for bioprocess development and operation.

## Abbreviations

ASCII American Standard Code for Information Interchanging

BDM Bacterial Dry Matter

cAMP cyclic adenosinemonophosphate

CFU Colony Forming Units

DC Dead Cells

DIGE differential -gel electrophoresis

FDA Food and Drug Administration

FSC Forward Light Scatter

GFP Green Fluorescent Protein

GUI Graphical User Interface

HPLC High Performance Liquid Chromatography

IPTG Isopropyl β-D-thiogalactopyranoside

OLE Object Linking and Embedding

OPC OLE for Process Control

PAT Process Analytical Technology

PCN Plasmid Copy Number

PLC Programmable Logic Controller

PLS Partial Least Squares – Model

ppGpp guanosine tetraphosphate

PTR Proton Transfer Reaction

qP Product formation rate

RBF Radial Basis Function – Network

RMSEP Root Mean Square Error of Prediction

RTKBS Real-Time Knowledge-Based System

SCADA Supervisory Control and Data Acquisition

SOM Self-Organising Map

SSC Side Light Scatter

TCN Total Cell Number
